# Artificial Intelligence for Personalized Perioperative Medicine

**DOI:** 10.7759/cureus.53270

**Published:** 2024-01-31

**Authors:** Elena Bignami, Matteo Panizzi, Valentina Bellini

**Affiliations:** 1 Department of Medicine and Surgery, Anesthesiology, Critical Care and Pain Medicine Division, Azienda Ospedaliero Universitaria di Parma, Parma, ITA

**Keywords:** patient's trajectories, internet of things (iot), transhumanism, perioperative medicine, artificial intelligence in anesthesia

## Abstract

The development of artificial intelligence (AI) is disruptive and unstoppable, also in medicine. Because of the enormous quantity of data recorded during continuous monitoring and the peculiarity of our specialty where stratification and mitigation risk are some of the core aspects, anesthesiology and postoperative intensive care are fertile fields where new technologies find ample room for expansion.

Recently, research efforts have focused on the development of a holistic technology that globally embraces the entire perioperative period rather than a fragmented approach where AI is developed to carry out specific tasks. This could potentially revolutionize the perioperative medicine we know today. In fact, AI will be able to expand clinician's ability to interpret, adapt, and ultimately act in a complex reality with facets that are too complex to be managed all at the same time and in a holistic manner.

With the support of new tools, as healthcare professionals we have the moral obligation to govern this transition, allowing an ethical and sustainable development of these technologies and avoiding being overwhelmed by them. We should welcome this transhumanist tension which does not aim at the replacement of human capabilities or even at the integration of these but rather at the expansion of a “single intelligence”.

## Editorial

The progress of artificial intelligence (AI) is disruptive and unstoppable. Let's think about the introduction of tools such as SpO2 or continuous invasive blood pressure monitoring into clinical practice: the application of AI could be even more impactful. Anesthesia and postoperative intensive care can provide the necessary fuel for the application of new technologies thanks to the production of large amounts of data, thus becoming fertile areas for their development [1].

The review by Singam [2] is an excellent summary of the current use of AI in perioperative medicine. In this paper, the author has achieved the objective of providing a complete overview of its applications from preoperative risk assessment to the postoperative phase. It is intriguing to note how both the applications and the applicability of AI in perioperative medicine have increased year by year: if we compare this review with those published a couple of years ago, we note that from a fragmented approach where AI was developed to carry out specific tasks (such as risk prediction or the prediction of specific events with the aim of creating clinical support tools) [3], a holistic technology is being developed which globally embraces the entire perioperative period and that could potentially revolutionize the perioperative medicine we know today, as described in the fascinating article by Feinstein et al. [4]. In the future, we will have the opportunity to use a machine learning-driven perioperative risk stratification interoperable with a control tower placed into the operating compartment and/or with the operating room anesthesiological machines and thus create a continuum between the preoperative and perioperative phases. Imagine a system that auto-recalibrates and adjusts the prediction in accordance with the new intraoperative parameters and then suggests the best postoperative monitoring strategy and setting: it will be like a support layer upon which different clinical support tools such as event prediction (e.g. hypotension prediction index, HPI), machine learning-derived outcome prediction models, target-controlled infusion, closed-loop anesthesia systems, or the medical early warning systems rely on. An AI fed in real time by other sub-AIs. The fundamental and challenging role of the clinician will be the right contextualization of the information obtained [5].

However, this speed of progression is not free from potential problems making the adaptation of human intelligence to these new technologies a particularly complex process. We know from biology that adaptation takes time, and this is a slower process than current technological evolution. There are three main problems related to its widespread adoption in clinical practice: the risk of the black box effect with consequent clinician’s knowledge atrophy, the insurance and medical-legal related issues, and the security and privacy-related issues. The solution for the latter will be the integration of blockchain-based systems: this is already possible [6,7], but their implementation must be compliant with complex medical-legal issues that should be first addressed. A great whitepaper that establishes an example of comprehensive general principles on this topic is the paper by Schnelldorfer et al. [7]. Certainly, the burden of adhering to their checklist cannot fall on the clinician: healthcare providers should have the role of promoting task forces on this topic that involve the engineering departments and forensic doctors, specialists in this field, and at the same time give the educational guidance to clinicians [8]. On the other hand, from this perspective, clinicians have the duty to focus on the first of the three problems described. The clinical practice must be always guided by the clinician’s comprehension of the relationship between the input given and the output provided by the AI tools. They must be developed in such a way that they could provide the possibility to re-construct the link between input and outputs so that it will be possible a real supervision [9].

What is now certain is that we cannot ignore the existence of AI. As described in the papers by Singam and Feinstein, AI is beginning to be seen as an extension and an enhancement of human capabilities [2,4]. In this context, it is impossible not to turn our gaze toward the transhumanist theories. AI will be able to expand man's ability to interpret, adapt, and ultimately act in a reality with facets that are too complex to be managed all at the same time and in a holistic manner. It is known from our everyday clinical practice that it is not always possible to have a clear overview of the relationship between all the variables with only mathematical models or our experience. This transhumanist tension does not aim to replace human capabilities or even to integrate them (e.g. with two "parallel" intelligences), but rather to enhance a single intelligence. This is the concept of the hybrid model [10]. Strictly speaking, AI does not "replace" because it performs tasks that a human would not be able to perform because of the quantity and complexity of the interactions between patient variables. Succeeding in managing such complexity will result in an improved quality of care. Plus reducing the clinician's cognitive overload will result in more safety for patients [5]. Let's think about the complex relationships between lifestyle, risk factors, drugs, and diseases, or the enormous amount of data deriving from a real-time collection of clinical data through the Internet of Things (IoT) and continuous remote monitoring: the "artificial part” acts as if it were an additional, almost external, part of our brain, working in the background and allowing us to automatically collect and elaborate the data, then the "human" one exploits this information by interpreting it and thus focusing on patient’s risk and his/her possible trends.

In perioperative medicine, this has the potential to enable us to comprehensively manage the pathophysiology of each specific patient and the possible implications on the outcome of our medical interventions through the simulation of trajectories. This is of paramount importance as our goal with surgery is to obtain a restitutio ab integrum of the preoperative patient functionality or at least approximate that state. To achieve this, it is necessary to know what this function is, to assess the risk of loss of function, and then to be able to decide with the patient what is the best perioperative care pathway, either on the basis of simulations and/or by intercepting or modulating their expectations as much as possible (e.g. by exploiting the advantages of patient-reported experience measures - PREMs). This may paradoxically increase the human touch that we inevitably risk losing if AI becomes substitutive or integrative.

The technology is developing so fast that in the near future, we could even create a digital twin of our patient. A non-fungible token (NFT) is a digital certificate that attests to the uniqueness, authenticity, and univocal ownership of a physical or digital object and all the relative information contained in it. These characteristics are guaranteed when registered on the blockchain: the tokenization of the health information of patients will guarantee the truthfulness and security of the data and control access to them thanks to encryption [6]. Ideally, this information can be accessed in a human-readable dashboard through software that visibly translates the information contained in these digital certificates as we exampled above. Imagine the possibility of having an NFT containing all of the patient's past information, including habits, risk factors, current functions, and attitudes, secured by blockchain tokenization. With such a transcendent copy of ourselves, we could ideally run real-time simulations for each possible path and estimate its impact on the patient's trajectory. Imagine a trajectory like a set of curves over time: these are the possible outcomes that a specific patient undergoing a certain kind of surgery tends to and are influenced by the patient's characteristics and perioperative complications. Identifying a bad trajectory in time may prevent a loss of function bringing back the patient on the curve of the restitutio ab integrum.

AI is bringing about a real technological revolution. As witnesses of this era and healthcare professionals, we have the moral obligation to govern this transition, allowing an ethical and sustainable development of these technologies and avoiding being overwhelmed by them. If managed well, these tools can lead us to an increasingly personalized perioperative medicine, capable of improving quality and safety for patients while enhancing the human side of our profession.
